# Procaspase-9 induces its cleavage by transnitrosylating XIAP via the Thioredoxin system during cerebral ischemia-reperfusion in rats

**DOI:** 10.1038/srep24203

**Published:** 2016-04-07

**Authors:** Dengyue Zhang, Ningjun Zhao, Bin Ma, Yan Wang, Gongliang Zhang, Xianliang Yan, Shuqun Hu, Tie Xu

**Affiliations:** 1 Jiangsu Key Laboratory of Brain Disease Bioinformation, Xuzhou Medical College, Xuzhou 221004, China; 2 Institute of Emergency Rescue Medicine, Xuzhou Medical College, Xuzhou 221002, China; 3 Emergency Center of the Affiliated Hospital of Xuzhou Medical College, Xuzhou 221002, China; 4 Department of Physiology, College of Basic Medical Science, Anhui Medical University, 81 Meishan Road, Hefei, Anhui 230032, China

## Abstract

Transnitrosylation is an important mechanism by which nitric oxide (NO) modulates cell signaling pathways. For instance, SNO-caspase-3 can transnitrosylate the X-linked inhibitor of apoptosis (XIAP) to enhance apoptosis. XIAP is a potent antagonist of caspase apoptotic activity. Decrease in XIAP activity via nitrosylation results in SNO-XIAP-mediated caspase activation. Considering the functional liaison of procaspase-9 and XIAP, we hypothesized that procaspase-9 nitrosylates XIAP directly. Our data confirmed that cerebral ischemia-reperfusion induced XIAP nitrosylation, procaspase-9 denitrosylation and cleavage. Interestingly, the time courses of the nitrosylation of procaspase-9 and XIAP were negatively correlated, which was more prominent after cerebral ischemia-reperfusion, suggesting a direct interaction. The nitrosylation of XIAP, as well as the denitrosylation and cleavage of procaspase-9, were inhibited by DNCB, TrxR1 AS-ODNs, or TAT-AVPY treatment. Meanwhile, DNCB, TrxR1 AS-ODNs, or TAT-AVPY also inhibited the decrease in hippocampal CA1 neurons induced by ischemia-reperfusion in rats. The denitrosylation and cleavage of procaspase-9 induced by OGD/reoxygenation in SH-SY5Y cells were inhibited when cells were co-transfected with wild-type procaspase-9 and XIAP mutant (C449G). These data suggest that cerebral ischemia-reperfusion induces a transnitrosylation from procaspase-9 to XIAP via the Trx system to consequently cause apoptosis. Additionally, Cys325 is a critical S-nitrosylation site of procaspase-9.

Caspase is a family of cysteine proteases that exists in metazoan cells in catalytically inactive zymogens known as procaspases. Once activated, the caspases cleave specific substrates next to aspartate or glutamate residue and induce cell apoptosis. The caspases can also be classified into two groups, namely, the upstream initiator caspases (e.g., caspase-2, -8, -9, and -10) and the downstream executioner caspases (e.g., caspase-3, -6, and -7)^1^. Effector caspases will cleave cellular substrates and induce apoptosis after cleaving by the initiator caspases, which, on the other hand, are activated by recruitment into large multi-protein complexes, such as the PIDDosome (casapase-2), the apoptosome (caspase-9) and the death inducing signaling complexes (DISCs) (caspase-8 and -10)^2^.

Cerebral ischemia triggers intrinsic and extrinsic caspase-dependent pathways of apoptosis^3^. During the intrinsic apoptosis induced by the mitochondria^4^, cell stress causes a disruption of the mitochondrial outer membrane which results in a release of cytochrome c from the intermembrane space into the cytosol. Cytochrome c then interacts with apoptotic protease activation factor-1 (APAF-1) to trigger nucleotide exchange and stepwise assembly of a heptameric apoptosome with procaspase-9 in a dATP/ATP dependent manner^5^,^6^. After activation through dimerization, procaspase initiates downstream executioner caspases^7^,^8^,^9^. Inhibitors for apoptosis (IAPs), such as X-linked inhibitor of apoptosis (XIAP)^10^, can inhibit procaspase activity. XIAP contains three baculovirus IAP repeats (BIRs) which can directly interact with caspases-3, -7, -9 and a C_3_HC_4_ RING finger motif, which plays a role of E3 ubiquitin ligase. Previous studies also demonstrate that ubiquitin-ligase activity is physiologically required for inhibition of caspases^11^,^12^.

Cerebral ischemia induces over stimulation of N-methyl-D-aspartate receptors (NMDARs), followed by excessive Ca^2+^ influx and activation of neuronal nitric oxide synthase (nNOS). NOS generates nitric oxide (NO) from L-arginine^13^,^14^,^15^ after binding to the postsynaptic density protein of 95 kDa (PSD-95); NO also regulates a variety of physiological and pathological processes across different biological systems^16^. The multifaceted actions in response to NO can be classified into authentic NO-mediated cGMP-dependent signalling and reactive nitrogen species-mediated cGMP-independent actions. Protein S-nitrosylation, in which NO reacts with the cysteine residues of target proteins to form S-nitrosothiols (SNOs), has emerged as a prototype of cGMP-independent and redox-dependent post-translational modifications^17^. Protein S-nitrosylation modulates neuronal survival and death; for example, S-nitrosylation of proteins [such as parkin, protein disulphide isomerase (PDI), glyceraldehyde-3-phosphate dehydrogenase (GAPDH), mixed lineage kinase 3 (MLK3) and XIAP] can cause neuronal damage and even cell death. In contrast, S-nitrosylation of caspases and NMDAR exhibits neuroprotective effects^18^,^19^,^20^. S-nitrosylation is a reversible post-translational modification and S-nitrosylated proteins can be denitrosylated by S-nitrosoglutathione reductase and the Thioredoxin (Trx) system^21^. In addition, Trx1 with SNO-Cys73 displays specific transnitrosylation properties, e.g., transferring NO from Cys73 to target proteins^22^.

Kim reported that S-nitrosylation of endogenous procaspase-9 in HT-29 cells occurs under normal conditions and denitrosylation of procaspase-9 enhances its cleavage and consequent apoptosis^23^. Furthermore, Nakamura and his colleagues found that the NO group was transferred from caspase-3 to XIAP in neurons under nitrosative or excitotoxic stress, resulting in XIAP S-nitrosylation and a decrease in antiapoptotic activity, with subsequent denitrosylation of caspase-3 and apoptotic cell death^24^. Accordingly, we examined whether procaspase-9 could transnitrosylate XIAP and enhance its cleavage and the consequent neuronal apoptosis during cerebral ischemia. Moreover, whether the Trx system mediates this transnitrosylation was explored in this study.

## Materials and Methods

### Antibody and reagents

Anti-caspase-9 (sc-70506, is used to detect procaspse-9), anti-XIAP (sc-11426), anti-TrxR1 (sc-20147), and anti-actin (sc-10731) were purchased from Santa Cruz Biotechnology (Santa Cruz, CA). Anti-caspase-9 (#9504, is used to detect cleaved caspase-9) was purchased from Cell Signaling Biotechnology (Boston, MA). The secondary anti-mouse IgG (A 1682) and anti-rabbit IgG (T 6778) were purchased from Sigma-Aldrich (St. Louis, MO). Dinitrochlorobenzene (DNCB) was obtained from Santa Cruz Biotechnology. Nitrosoglutathione (GSNO), Neocuproine, methyl methanethiosulfonate (MMTS) and Ascorbate Streptavidin-agarose were obtained from Sigma. Lipofectamine^TM^2000 was obtained from Life Technologies (Carlsbad, CA). Biotin-HPDP was obtained from Millipore (Billerica, MA). The RT-PCR primers used to amplify XIAP, mutant XIAP and procaspase-9 plasmids, Trx1 antisense oligodeoxynucleotides (AS-ODNs) and missense oligodeoxynucleotides (MS-ODNs) were synthesized by the Shanghai Sangon Biological Engineering Technology and Services Co., Ltd. (Shanghai, China). The peptides H-YGRKKRRQRRR-AVPY-OH (Tat-AVPY) and H-YGRKKRRQRRR-MMLL-OH (Tat-MMLL) were synthesized by the Shanghai Apeptide Co., Ltd (Shanghai, China).

### Plasmids

The expression plasmid (pCMS-EGFP-procaspase-9) encoding rat full-length caspase-9 was a kind gift of Dr. James M. Angelastro (Department of Molecular Biosciences, UC Davis School of Veterinary Medicine, Davis, CA). The pcDNA3.1-XIAP plasmid was constructed as follows: Total RNA was isolated from a fresh rat hippocampus. The XIAP insert was amplified by RT-PCR. The PCR product and the pcDNA3.1 vector were then digested and ligated to generate pcDNA3.1- XIAP.

### Site-directed mutagenesis of procaspase-9 and XIAP

Mutant procaspase-9 plasmids were constructed using the pCMS-EGFP-procaspase-9 plasmid as a template. Each cysteine (C) residue of procaspase-9 was mutated to glycine (G). The forward and reverse primers used to introduce mutations are as follows: C12G, CAACTCCTGCGGCGAGGCAGGGTACGCCTTG (forward) and CAAGGCGTACCCTGCCTCGCCGCAGGAGTTG (reverse); C76G, CCTCGCTTCATCTCCGGCTTAGAGGACACAGG (forward) and CCTGTGTCCTCTAAGCCGGAGATGAAGCGAGG (reverse); C178G, GCAGAGCTCATGATGTCGGTACTCCAGGGAAGATC (forward) and GATCTTCCCTGGAGTACCGACATCATGAGCTCTGC (reverse); C198G, CTGGACTCGGATCCCGGTGGCCACTGCCTC (forward) and GAGGCAGTGGCCACCGGGATCCGAGTCCAG (reverse); C201G, GATCCCTGTGGCCACGGCCTCATCATCAAC (forward) and GTTGATGATGAGGCCGTGGCCACAGGGATC (reverse); C210G, CAACAACGTGAACTTCGGCCCTTCCTCAGGGC (forward) and GCCCTGAGGAAGGGCCGAAGTTCACGTTGTTG (reverse); C225G, GCTCCCACGTGGACGGTGAGAAGCTTCAG (forward) and CTGAAGCTTCTCACCGTCCACGTGGGAGC (reverse); C233G, GCTTCAGCACCGCTTCGGCTGGCTGCGCTTC (forward) and GAAGCGCAGCCAGCCGAAGCGGTGCTGAAGC (reverse); C267G, CACCGTGCCCTGGACGGCTTCGTGGTGGTC (forward) and GACCACCACGAAGCCGTCCAGGGCACGGTG (reverse); C277G, CTCTCTCATGGCGGCCAGGCCAGCC (forward) and GGCTGGCCTGGCCGCCATGAGAGAG (reverse); C294G, CTATGGCACAGATGGAGGCTCTGTGTCCATCG (forward) and CGATGGACACAGAGCCTCCATCTGTGCCATAG (reverse); C310G, CAATGGGACCGGCGGCCCCAGCCTGG (forward) and CCAGGCTGGGGCCGCCGGTCCCATTG (reverse); C325G, CTTCATCCAGGCCGGTGGTGGTGAGCAG (forward) and CTGCTCACCACCACCGGCCTGGATGAAG (reverse); C441G, GCAGATTCCTGGCGGTTTTAACTTCCTCC (forward) and GGAGGAAGTTAAAACCGCCAGGAATCTGC (reverse); Mutant XIAP (C449G) was constructed using the pcDNA3.1-XIAP plasmid as a template with the forward primer: GCCTACAAGAGGAGAAGCTTGGCAAAATCTGTATGGATAG and the reverse primer: CTATCCATACAGATTTTGCCAAGCTTCTCCTCTTGTAGGC. All mutant plasmids were constructed using the QuikChange II kit (Stratagene, La Jolla, CA). Mutagenesis was confirmed by automated nucleotide sequencing.

### Cell culture and plasmid transfection analysis

293T cells were grown in Dulbecco’s modified Eagles medium (DMEM; Life Technologies, Carlsbad, CA) supplemented with 10% fetal calf serum (37 °C, 5% CO_2_). Wild-type (WT) or mutant-type (MT) pCMS-EGFP-procaspase-9 plasmids were transfected into 293T cells using lipofectamine 2000 in accordance with the manufacturer’s instruction (achieving a 70–80% transfection efficiency as determined by EGFP labeling). After transfection, cells were cultured at 37 °C with 5% CO_2_ for 24 h before 1 h of 200 μM GSNO treatment. Transfected cells were then harvested and homogenized in ice-cold HEN buffer containing 250 mM HEPES-NaOH, pH 7.7, 1 mM EDTA, and 0.1 mM neocuproine. The homogenates were centrifuged at 13,000 g for 15 min at 4 °C. Supernatants were collected, and protein concentrations were determined with the Bicinchoninic Acid assay (Beyotime Biotechnology, China). Samples were stored at −80 °C until use. To further test the transnitrosylation between procaspase-9 and XIAP, SH-SY5Y cells were transiently co-transfected with pCMS-EGFP-procaspase-9 and pCDNA3.1-XIAP plasmids in a 1:1 ratio. After 24 h, glucose free Earle’s balanced salt solution (EBSS) medium supplemented with gentamycin was purged with N_2_/CO_2_ (95%/5%) for 20 min, resulting in an oxygen content of 1%. Transfected SH-SY5Y cells were then washed three times with the same medium and incubated in an oxygen-free N_2_/CO_2_ (95%/5%) environment without oxygen for 3.5 h.

### Ischemia development and drug treatments

Adult male Sprague-Dawley rats (200–250 g, purchased from Shanghai Experimental Animal Center, Chinese Academy of Science, Shanghai, China) were used. All animal experiments were conducted and approved by the Institutional Animal Care and Use Committee of Xuzhou Medical College and the methods were carried out in accordance with approved guidelines (Assurances No. 2015 – 46, 2015-47). Transient cerebral ischemia was induced using the four-vessel occluded (4-VO) method as described previously^25^. Briefly, rats were anesthetized with chloral hydrate (300 mg/kg, intraperitoneally) before bilateral vertebral arteries were occluded permanently by electrocautery. Left and right carotid arteries were isolated. Then rats were allowed to recover for 24 h, before bilateral carotid arteries were occluded with aneurysm clips to induce cerebral ischemia without chloral hydrate pretreatment. After 15 min occlusion, the aneurysm clips were removed to allow reperfusion. Rats lost their righting reflex within 30 s and pupil light reflex were selected for the following experiments. Rectal temperature was maintained at 36.5–37.5 °C during the ischemia (15 min) and reperfusion process. Sham control rats received the same surgical procedures except that the carotid arteries were not occluded. Tat-AVPY and Tat-MMLL (400 μg/kg) dissolved in 0.9% NaCl and DNCB (240 μg/kg) dissolved in 1% dimethyl sulfoxide (DMSO) were intracerebroventricularly injected 40 min before ischemia. 10 nmol of Trx1 AS-ODNs and MS-ODNs in 10 μl TE buffer (10 mM Tris-HCl, pH 8.0, 1 mM EDTA) were administered every 24 h for 3 days. Control rats were intracerebroventricularly given corresponding solvent (1% DMSO or TE buffer).

### Sample preparation

Rats were decapitated immediately after reperfusion at the specified time, and the hippocampal CA1 region was isolated and frozen immediately in liquid nitrogen. These tissues were then homogenized in an ice-cold buffer containing 50 mM MOPS (pH 7.4), 100 mM KCl, 320 mM sucrose, 50 mM NaF, 0.5 mM MgCl_2_, 0.2 mM DTT (except when S-nitrosylation was tested), 1 mM EDTA, 1 mM EGTA, 1 mM Na_3_VO_4_, 20 mM sodium pyrophosphate, 20 mM phosphoglycerol, 1 mM p-nitrophenyl phosphate, 1 mM benzamidine, 1 mM phenylmethylsulfonyl fluoride, 5 μg/ml leupeptin, 5 μg/ml aprotinin, and 5 μg/ml pepstatin A. The homogenates were then sonicated for 3 bursts (10 s each) using a 3 mM probe of a Sanyo Soniprep 150 Ultrasonic disintegrator at an amplitude setting of 10 μm, followed by centrifugation at 13,000 g for 15 min at 4 °C. Supernatants were collected carefully, and the protein concentration was determined by Bicinchoninic Acid assay. Samples were aliquoted and stored at −80 °C until use.

### Determination of protein S-nitrosylation

Protein S-nitrosylation was detected by a biotin switch assay as described previously by Jaffrey under light-free conditions and using opaque tubes^26^, with some modifications. Briefly, the cells or hippocampi were homogenized in HEN buffer containing 250 mM HEPES-NaOH, pH 7.7, 1 mM EDTA, and 0.1 mM neocuproine. Free thiols were blocked by methylation with methyl methanethiosulfonate (MMTS). Unreacted methyl methanethiosulfonate was removed by protein precipitation in 10 volumes of acetone (−20 °C). Cysteine residues that had been S-nitrosylated were converted to free thiols with sodium ascorbate (1 mM final concentration), which does not alter the methylated thiols. The free thiols were then biotinylated with biotin-hexyl pyridyldithiopropionamide (HPDP) at 25 °C for more than 1 h. Thus, the S-nitrosylated cysteines were switched for biotin. Proteins were precipitated by chilled acetone, and the pellet was resuspended in HENS buffer (250 mM Hepes, pH 7.7, 1 mM EDTA, 0.1 mM neocuproine, 1% SDS). Biotinylated proteins were precipitated with streptavidin agarose and eluted from the beads with a solution containing 20 mM Hepes–NaOH, pH 7.7, 100 mM NaCl, 1 mM EDTA and 100 mM β-mercaptoethanol.

### Immunoprecipitation and Western blot

For immunoprecipitation, the homogenized samples (400 μg of protein) were diluted four-fold with immunoprecipitation buffer (50 mM Hepes buffer, pH 7.4, containing 10% glycerol, 150 mM NaCl, 1% Triton X-100, 0.5% Nonidet P-40, 1 mM EDTA, 1 mM EGTA, 1 mM phenylmethylsulfonyl fluoride, and 1 mM Na_3_VO_4_). Samples were preincubated for 1 h with 20 μl of protein A-sepharose CL-4B (Amersham Biosciences, Uppsala, Sweden) at 4 °C and centrifuged to remove proteins that had adhered non-specifically to protein A. The supernatants were then incubated with 1–2 μg of primary antibodies for 4 h or overnight at 4 °C. Protein A was added to the tube for a further 2 h incubation. Samples were then centrifuged at 10,000 g for 2 min at 4 °C and the pellets were washed three times with immunoprecipitation buffer. Bound proteins were eluted by boiling at 100 °C for 5 min in SDS-PAGE loading buffer and then isolated by centrifugation. The supernatants were separated on polyacrylamide gels and then electrotransferred to a PVDF membrane (Amersham Biosciences, Buckinghamshire, UK). After blocking for 3 h in Tris-buffered saline with 0.1% Tween 20 (TBST) and 3% bovine serum albumin, membranes were incubated overnight at 4 °C with primary antibodies in TBST containing 1% bovine serum albumin. The filters were then washed and incubated with alkaline phosphatase-conjugated secondary antibodies in TBST for 2 h and developed using NBT/BCIP color substrate (Promega, Madison, WI). The density of the bands on the membrane was scanned and analyzed with LabWorks image analysis software (UVP, Inc.).

When necessary, to examine protein S-nitrosylation, sample were added protein 2 × sodium dodecyl sulfate (SDS) sample buffer (100 mM Tris-HCl (pH6.8), 4% SDS, 0.2% bromophenol blue, and 20% glycerol) without the reducing agent DTT or β-mercaptoethanol. After boiling for 5 min, the samples were separated by SDS-PAGE and transferred to PVDF membranes.

### Histology

Rats were intracardial perfused with 4% paraformaldehyde in 0.1 M sodium phosphate buffer (pH 7.4) under deep anesthesia. Brains were removed quickly and further fixed with the same fixation solution overnight at 4 °C. Post-fixed brains were embedded in paraffin, followed by preparation of coronal sections of 6 μm thickness using a microtome. The paraffin-embedded brain slices were deparaffinized with xylene and rehydrated with ethanol at graded concentrations of 100–70% (v/v), followed by water washing. Brain tissue sections were stained with 0.1% (w/v) cresyl violet and examined by light microscopy. The number of surviving hippocampal CA1 pyramidal cells (1 mM length) was counted as the neuronal density.

### Data analysis and statistics

Values are expressed as the means ± SD. One-way analysis of variance (ANOVA), followed by the Duncan’s new multiple range method or the Newman-Keuls test was used. *P* values of <0.05 were considered significant.

## Results

### The Cys325 residue of procaspase-9 is a critical S-nitrosylation site

**Figure 1:**
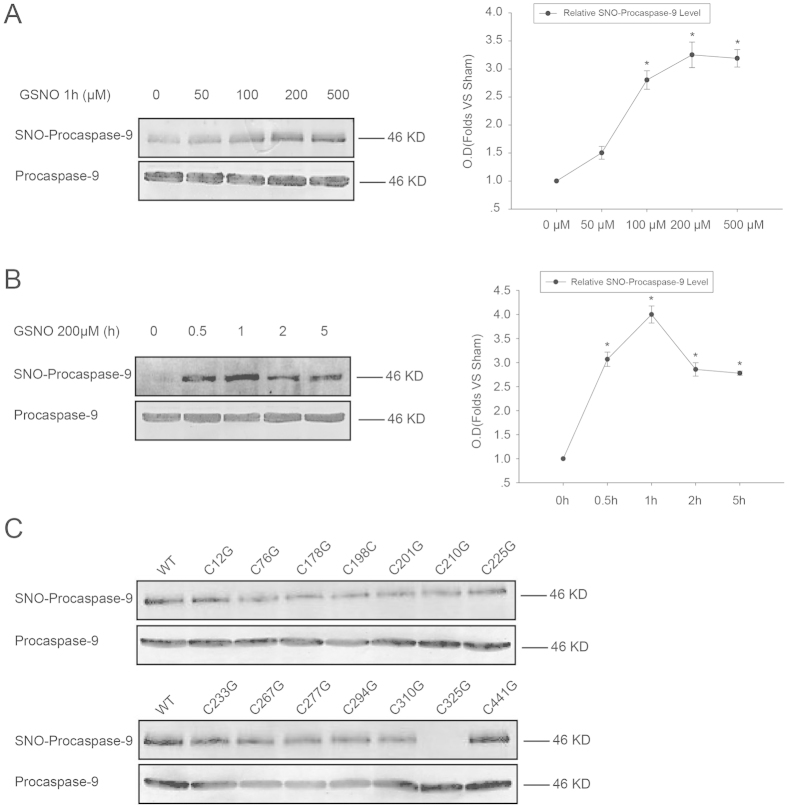
Identification of Cys325 as the S-nitrosylated cysteine residue of procaspase-9. (**A**) 293T cells expressing procaspase-9 were incubated with various concentrations of GSNO for 1 h and assayed for SNO-procaspase-9. Bands were quantified, and the intensities were represented as fold-increase vs. 0 μM groups. Data were expressed as means ± S.D. from three independent experiments (n = 3). *P < 0.05 vs. 0 μM groups. (**B**) Time course of the SNO-procaspase-9 level in transfected 293T cells treated with 200 μM GSNO. Bands were quantified, and the intensities were presented as fold-increase vs. 0 h groups. Data were expressed as means ± S.D. from three independent experiments (n = 3). *P < 0.05 vs. 0 h groups. (**C**) 293T cells overexpressing WT or mutant procaspase-9 were exposed to 200 μM GSNO for 1 h and SNO-procaspase-9 was detected using the biotin-switch assay.

First, we tested whether procaspase-9 can be S-nitrosylated by the exogenous nitric oxide donor GSNO. 293T cells transfected with pCMS-EGFP-procaspase-9 were then treated 24 h later with GSNO at different concentrations (0 μΜ, 50 μΜ, 100 μΜ, 200 μΜ, and 500 μΜ) for 1 h. SNO-procaspase-9 was visualized by a biotin-switch assay. As shown in Fig. 1A, GSNO markedly enhanced the levels of S-nitrosylated procaspase-9 and the 200 μM GSNO produced the strongest reaction. Then, 293T cells transfected with pCMS-EGFP-procaspase-9 for 24 h were treated with 200 μM GSNO for different intervals (0 h, 0.5 h, 1 h, 2 h, and 5 h), and the result showed that 1 h of 200 μΜ GSNO treatment was the optimal condition for S-nitrosylation of procaspase-9 (Fig. 1B). To further determine the target cysteine residue(s) that are sensitive to GSNO-induced S-nitrosylation of procaspase-9, we constructed 14 versions of procaspase-9, each a single-point cysteine mutant (harboring a cysteine to glycine mutation), and tested each for its S-nitrosylation induced by GSNO (200 μΜ for 1 h) using a biotin-switch assay. The result showed that the S-nitrosylation of the C325G mutant, but not the rest, was decreased significantly as compared with the wild type (Fig. 1C).

### SNO-procaspase-9 transnitrosylates XIAP and contributes to its cleavage during cerebral ischemia-reperfusion

**Figure 2:**
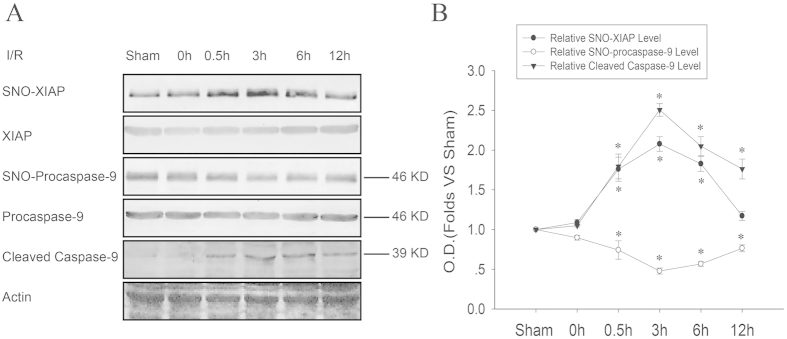
The time course of transnitrosylation from procaspase-9 to XIAP, and the cleavage of procaspase-9 induced by ischemia-reperfusion (I/R). (**A**) The time course of S-nitrosylated XIAP and procaspase-9, and the cleavage of procaspase-9 (cleaved caspase-9) in the rat hippocampal CA1 derived from sham-treated animals or rats subjected to 15 min ischemia at various time points after reperfusion. (**B**) Bands were quantified, and the intensities were presented as fold-increase vs. the sham group. Data were the means ± S.D. from four independent experiments (n = 4). *P < 0.05 vs. the sham group.

Cerebral ischemia promotes the cleavage of procaspase-9 and subsequent apoptosis. To investigate the role of S-nitrosylation in this process, we examined the time course of the S-nitrosylation of both procaspase-9 and XIAP, and the cleavage of procaspase-9. As shown in Fig. 2, procaspase-9 was denitrosylated significantly at 0.5, 3, 6, and 12 h of reperfusion, especially at 3 h reperfusion. Interestingly, XIAP was S-nitrosylated significantly at 0.5, 3, and 6 h of reperfusion, especially at 3 h. The cleavage of procaspase-9 showed a similar tendency of denitrosylation as procaspase-9. These results suggest a possibility that SNO-procaspase-9 transnitrosylates XIAP before its cleavage.

### Disrupting the interaction between XIAP and procaspase-9 attenuates transnitrosylation during cerebral ischemia-reperfusion

**Figure 3:**
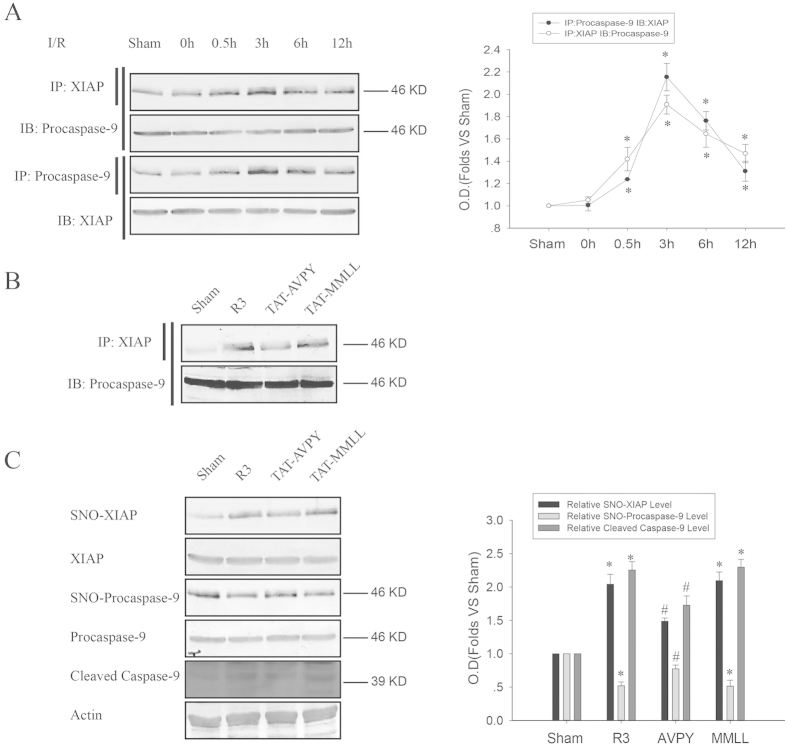
The effects of the interaction of XIAP with procaspase-9 on the transnitrosylation from procaspase-9 to XIAP and the cleavage of procaspase-9 induced by I/R. The time course of interaction was detected using immunoprecipitation. (**A**) Sample proteins were immunoprecipitated (IP) with anti-XIAP antibody followed by immunoblot (IB) with anti-procaspase-9 antibody, and IP with anti- procaspase-9 antibody, followed by IB with anti-XIAP antibody. Bands were quantified, and the intensities were represented as fold-increase vs. the sham group. Data were the means ± S.D. from four independent experiments (n = 4). *P < 0.05 vs. the sham group. (**B**) Rats were treated with TAT-AVPY and TAT-MMLL. Sample proteins were immunoprecipitated (IP) with anti-XIAP antibody, followed by immunoblot (IB) with anti-procaspase-9 antibody. (**C**) Rats were treated with TAT-AVPY and TAT-MMLL to observe their effects on the S-nitrosylation of XIAP and procaspase-9, and the cleavage of procaspase-9 (cleaved caspase-9). Bands were quantified, and the intensities presented as fold-increase vs. the sham group. Data were the means ± S.D. from four independent experiments (n = 4). *P < 0.05 vs. the sham group, ^#^*P* < 0.05 vs. the R3 group.

Transnitrosylation between proteins usually requires an interaction of the two proteins. Considering this, we used imunoprecipitation to test whether XIAP interacts with procaspase-9 during cerebral ischemia-reperfusion. As shown in Fig. 3A, the interaction of XIAP with procaspase-9 was enhanced after 0.5, 3, 6, or 12 h reperfusion with a peak level at 3 h. To further determine whether this interaction is necessary for the transnitrosylation of SNO-procaspase-9 into XIAP, we designed a peptide YGRKKRRQRRRAVPY (TAT-AVPY; AVPY is a conserved Smac-like IAP binding tetra-peptide motif of procaspase-9^27^ and TAT encoded by HIV-1 is the small basic protein used to deliver cross-linked proteins from the extracellular space to the cytosol). The peptide was injected intracerebroventricularly 40 min before ischemia to competitively inhibit the interaction between XIAP and procaspase-9. As shown in Fig. 3B, TAT-AVPY, but not the control peptide TAT-MMLL, significantly inhibited the interaction between XIAP and procaspase-9. We further examined whether TAT-AVPY affects the transnitrosylation from procaspase-9 to XIAP using the biotin-switch assay. As shown in Fig. 3C, TAT-AVPY remarkably inhibited the denitrosylation of procaspase-9 and the S-nitrosylation of XIAP and the cleavage of procaspase-9 in rats that received I/R (ischemia/reperfusion) for 3 h; the control peptide TAT-MMLL did not present such an inhibition. These results further confirm that SNO-procaspase-9 transnitrosylates XIAP to facilitate its cleavage during cerebral ischemia-reperfusion.

### The Thioredoxin 1 (Trx1) system mediates the transnitrosylation from procaspase-9 to XIAP during cerebral ischemia-reperfusion

**Figure 4:**
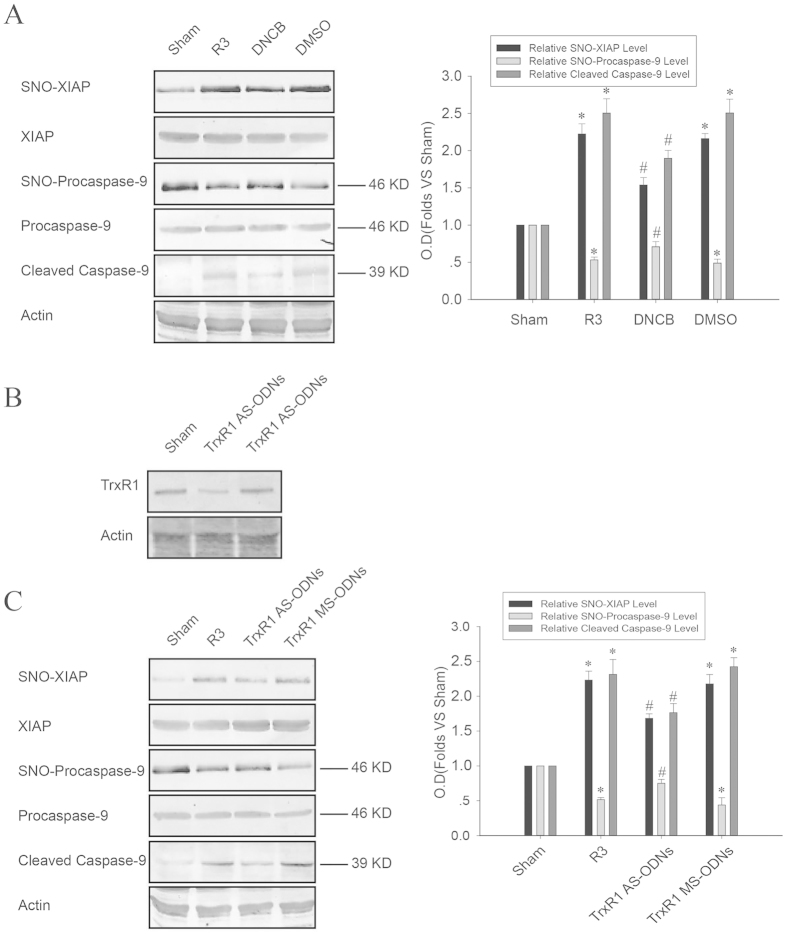
Effects of DNCB and TrxR1 AS-ODNs on the transnitrosylation from procaspase-9 to XIAP, and the cleavage of procaspase-9 induced by I/R. (**A,C**) Rats were treated with DNCB, DMSO, TrxR1 AS-ODNs and TrxR1 MS-ODNs to observe their effects on the S-nitrosylation of XIAP and procaspase-9, as well as the cleavage of procaspase-9 (cleaved caspase-9). Bands were quantified, and the intensities were presented as fold-increase vs. sham. Data were the means ± S.D. from four independent experiments (n = 4). *P < 0.05 vs. the sham group, ^#^*P* < 0.05 vs. the R3 group. (**B**) Rats were treated with TrxR1 AS-ODNs and TrxR1 MS-ODNs; TrxR1 was detected using Western blot.

Previous studies have shown that Trx1 possesses either a transnitrosylation or a denitrosylation activity toward target proteins^22^. Thus, we determined to elucidate whether the Trx1 system mediates the transnitrosylation from procaspase-9 to XIAP. First, DNCB, a highly potent and selective irreversible inhibitor of TrxR1, was given to rats 20 min before ischemia through intracerebroventricular injection. Then, the biotin-switch assay and immunoblotting were performed to examine the S-nitrosylation of procaspase-9 and XIAP, as well as the cleavage of procaspase-9. As shown in Fig. 4A, pretreatment with DNCB significantly suppressed the denitrosylation of procaspase-9, the S-nitrosylation of XIAP and the cleavage of procaspase-9. Next, we investigated the influence of the Trx1 system in this process by TrxR1 knockdown (Fig. 4B). As shown in Fig. 4C, TrxR1 AS-ODNs but not TrxR1 MS-ODNs downregulated TrxR1 expression and showed an analogous effect to DNCB on the S-nitrosylation of procaspase-9 and XIAP and the cleavage of procaspase-9. These results demonstrate that the Trx1 system is essential to the transnitrosylation from procaspase-9 to XIAP during cerebral ischemia-reperfusion.

### The neuroprotective effect of drugs preventing transnitrosylation from procaspase-9 to XIAP

**Figure 5:**
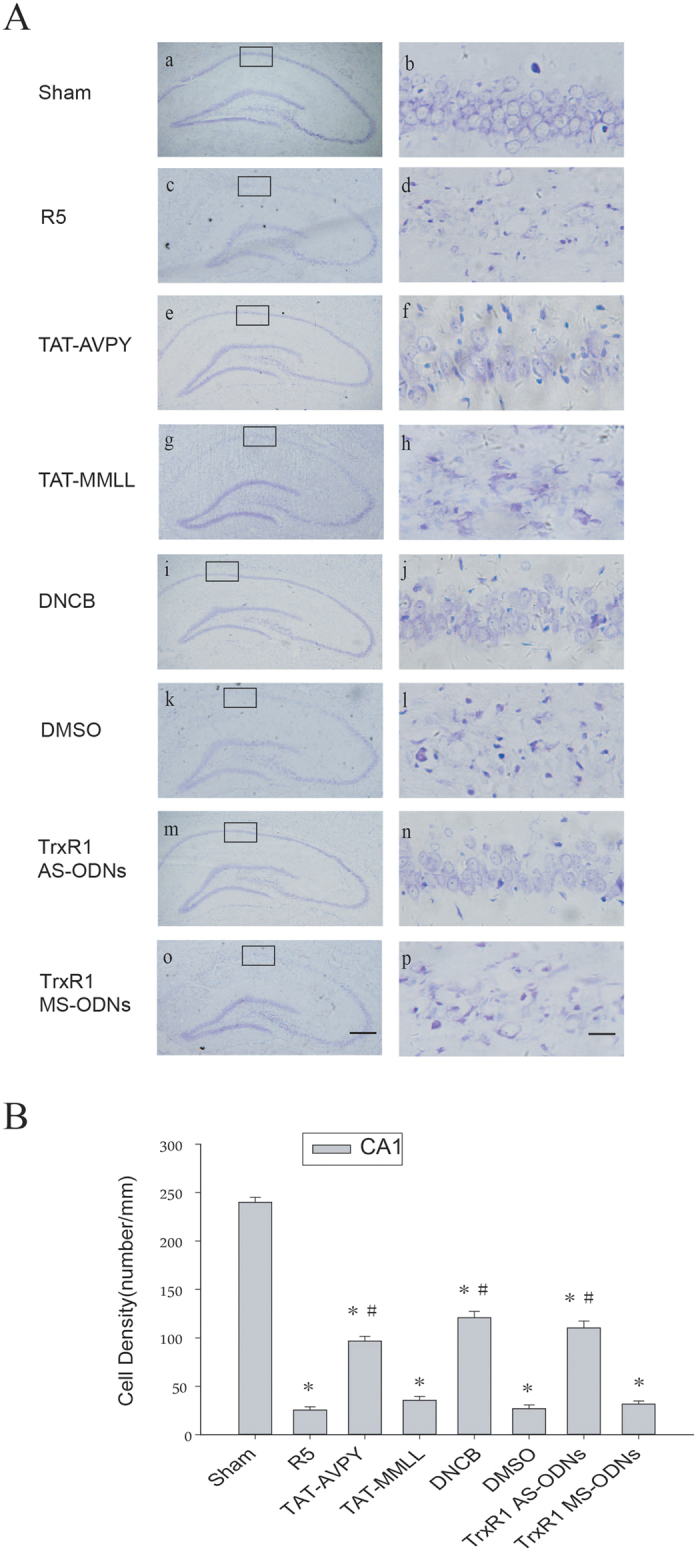
The neuroprotection effect of DNCB, TrxR1 AS-ODNs and TAT-AVPY against injury in rat hippocampal CA1 regions induced by I/R. (**A**) Cresyl violet staining was performed on sections of rat hippocampi subjected to sham operation (a and b), five days of reperfusion after global ischemia (c and d), or pretreatment with TAT-AVPY (e and f), DNCB (i and j), TrxR1 AS-ODNs (m and n), TAT-MMLL (g and h), DMSO (k and l), or TrxR1 MS-ODNs (o and p). The boxed areas in the left column are shown at higher magnification in the right columns. Original magnification ×40 in panels a, c, e, g, i, k, m and o; and ×400 in panels b, d, f, h, j, l, n and p. Scale bars: 200 μm (panels a, c, e, g, i, k, m and o) and 10 μm (panels b, d, f, h, j, l, n and p). (**B**) The cell density was expressed as the number of cells per 1 mM length of the CA1 pyramidal cells counted under a light microscope. Data were the mean ± S.D. (n = 6). *P < 0.05 vs. the sham group, ^#^*P* < 0.05 vs. the R5d group.

As previously mentioned, pretreatment with TAT-AVPY, DNCB or TrxR1 AS-ODNs attenuated the transnitrosylation from procaspase-9 to XIAP and the cleavage of procaspase-9. Therefore, Cresyl violet staining was used to test whether these drugs exhibit a neuroprotective effect against the apoptosis induced by cerebral ischemia-reperfusion in the hippocampal CA1 region. Normal neurons present round and pale stained nuclei, whereas shrunken cells with pyknotic nuclei were regarded as dead ones. As shown in Fig. 5, the sham operation group showed normal neurons, whereas 5 d of reperfusion following 15 min of cerebral ischemia resulted in severe cell death. Rats pretreated with TAT-AVPY, DNCB or TrxR1 AS-ODNs showed a dramatic decrease in neuronal degeneration. However, pretreatment with TAT-MMLL, DMSO or TrxR1 MS-ODNs failed to show any neuroprotective effects.

### Transnitrosylation from procaspase-9 to XIAP promotes the cleavage of procaspase-9 induced by OGD/reoxygenation in SH-SY5Y cells

**Figure 6:**
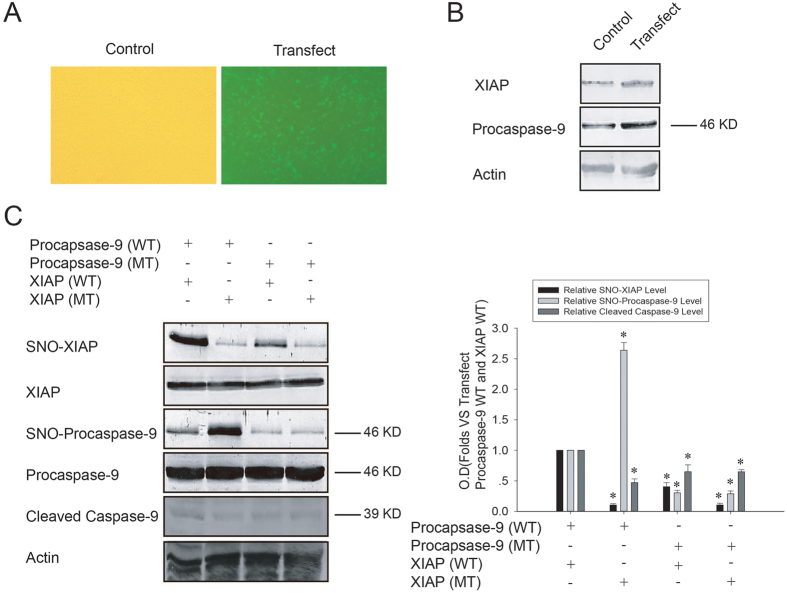
Effects of transnitrosylation from procaspase-9 to XIAP on the cleavage of procaspase-9 induced by OGD/reoxygenation. (**A,B**) SH-SY5Y cells were cotransfected with pCMS-EGFP-procaspase-9 (WT) and pcDNA3.1-XIAP (WT). After 24 h, green fluorescence was observed under fluorescent microscopy, and overexpressed procaspase-9 and XIAP were detected using Western blot. (**C**) SH-SY5Y cells were cotransfected with pCMS-EGFP-procaspase-9 and pcDNA3.1-XIAP; after 3.5 h of OGD followed by 6 h of reoxygenation, the S-nitrosylation of XIAP and procaspase-9 was detected using a biotin switch assay, and cleaved caspase-9 was detected using Western blot. Bands were scanned, and the intensities were represented as fold-increase vs. cotransfected with wild type pCMS-EGFP-procaspase-9 and wild type pcDNA3.1-XIAP groups. Data were shown as means ± S.D. from three independent experiments (n = 3). *P < 0.05 vs. the cotransfected with wild type pCMS-EGFP-procaspase-9 and the wild type pcDNA3.1-XIAP groups.

To further confirm the effect of transnitrosylation from procaspase-9 to XIAP on the cleavage of procaspase-9, we employed SH-SY5Y cells co-transfected with pCMS-EGFP-procaspase-9 and pcDNA3.1-XIAP followed by OGD/reoxygenation treatment. As shown in Fig. 6A,B, co-transfection with pCMS-EGFP-procaspase-9 and pcDNA3.1-XIAP significantly increased the procaspase-9 and XIAP expression. As shown in Fig. 6C, 3.5 h OGD treatment followed by 6 h reoxygenation resulted in more cleavage of procaspase-9 in SH-SY5Y cells co-transfected with wild-type procaspase-9 and wild type XIAP than those co-transfected with wild type procaspase-9 and mutant XIAP (C449G). We simultaneously detected the S-nitrosylation of procaspase-9 and XIAP. As shown in Fig. 6C, the S-nitrosylation level of XIAP was significantly higher in cells co-transfected with wild-type procaspase-9 and XIAP than in those co-transfected with wild-type procaspase-9 and mutant XIAP (C449G), but the S-nitrosylation level of procaspase-9 was opposite to that of XIAP between these two groups. Cys449 is a known S-nitrosylation site of XIAP^28^, where XIAP with the Cys449 mutation to glycine loses the ability to be transnitrosylated. These results confirm that transnitrosylation from procaspase-9 to XIAP can promote the cleavage of procaspase-9.

## Discussion

**Figure 7:**
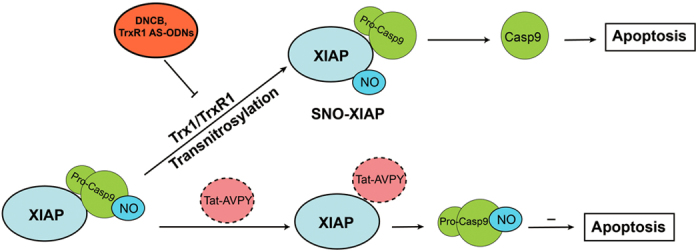
Cerebral ischemia-reperfusion induces procaspase-9 (Pro-Casp9) transnitrosylate XIAP, enhance the cleavage of procaspase-9 (Casp9) and the consequent neuronal apoptosis. The transnitrosylation is inhibited by DNCB, TrxR1 AS-ODNs, or TAT-AVPY treatment.

In this study, we for the first time reveal that SNO-procaspase-9 transnitrosylates XIAP, contributing to its cleavage during cerebral ischemia-reperfusion, and that the transnitrosylation occurs between procaspase-9 and XIAP. We further demonstrate that the Trx1 system mediates this transnitrosylation through inhibiting TrxR1 (DNCB) activity or downregulating TrxR1 (TrxR1 AS-ODNs) expression (Fig. 7). Additionally, overexpressed procaspase-9 can be S-nitrosylated at cysteine 325 by an exogenous NO donor GSNO *in vitro*. Considering that transfection of XIAP with mutated S-nitrosylation residue attenuates the cleavage of procaspase-9 induced by OGD/reoxygenation in SH-SY5Y cells, we confirm that transnitrosylation from procaspase-9 to XIAP plays an essential role during the apoptosis induced by cerebral ischemia-reperfusion or OGD/reoxygenation.

The best-characterized S-nitrosylation residue(s) of the caspase family of proteases is the Cys163 of caspase-3 which is involved in the conserved sequence QACRG, an essential structure mediating the catalytic activity of caspase-3. S-nitrosylation of the active site Cys163 leads to an inhibition of caspase-3 activity and apoptosis^29^. However, the S-nitrosylation residue(s) of procaspase-9 remains unidentified. Here, we identified Cys325 as a critical S-nitrosylation residue of procaspase-9 through a Site-directed mutagenesis study. Interestingly, Cys325 is also in the middle of the conserved sequence (QACGG) mediating the catalytic activity of procaspase-9. Hence, we speculate that S-nitrosylation of the active site may modulate the catalytic activity of the rest of the members of the caspase family.

As a redox-based post-translational modification of specific cysteine thiol side chains by nitric oxide, S-nitrosylation is a common phenomenon seen in signal transduction^21^, similar to phosphorylation regulation^30^. Recent years have witnessed many new functions of denitrosylation and transnitrosylation in regulating protein S-nitrosylation. The Thioredoxin (Trx) system–which consists of Thioredoxin (Trx) proteins, Thioredoxin reductase (TrxR) proteins and nicotinamide adenine dinucleotide phosphate (NADPH)–contains important denitrosylating enzymes involved in both physiological and pathological conditions^21^,^31^. Both cytosolic and mitochondrial Thioredoxins regulate basal and FAS-induced denitrosylation of caspase-3 in mammalian cells^31^. Trx1 is implicated in the denitrosylation of endothelial nitric oxide synthase (eNOS), which contributes to its homodimerization and activation *in vitro*^28^. The Thioredoxin-1 (Trx1) system mediates N-Methyl-D-Aspartate Receptor (NMDAR)-dependent denitrosylation via neuronal nitric oxide synthase (nNOS) during the early stage of cerebral ischemia/reperfusion by increasing the enzyme activity^32^. Trx has also been reported to act as a transnitrosylation agent, by transnitrosylating procaspase-3 to inhibit the apoptosis induced by etoposide in Jurkat cells^33^. In line with this process, Wu and colleagues have identified 47 putative Trx1-mediated transnitrosylation target proteins in transnitrosylation-active Trx1C32S/C35S expressing HeLa cells^34^.

The reverse trend of the time course of the S-nitrosylation of procaspase-9 and XIAP suggests the possibility that procaspase-9 transnitrosylates XIAP during cerebral ischemia-reperfusion. Transnitrosylation needs direct protein-protein interaction, and our results also confirmed this point, as shown in Fig. 3. Whether XIAP can directly interact with procaspase-9 remain elusive, as earlier literature suggested a possibility^35^,^36^, but recent studies emphasize that XIAP can only interact directly with processed capase-9 in either human U-937 myeloid leukemia cells stably overexpressing XIAP or reconstituted caspase-9-Apaf-1 holoenzyme complexes containing fully processed caspase-9 or unprocessed procaspase-9^27^,^37^. However, Kim and Tannenbaum found that XIAP was able to bind the precursor of caspase-9^38^. In this study, we demonstrated that cerebral ischemia-reperfusion enhanced the interaction between procaspase-9 and XIAP, and pretreatment with TAT-AVPY [which can competitively bind the third baculoviral IAP repeat (BIR3) domain of XIAP] significantly inhibited this interaction (Fig. 7). So, our results provide new evidence for the direct interaction between procaspase-9 and XIAP. In addition, this interaction is essential for procaspase-9 to transnitrosylate XIAP.

XIAP is an anti-apoptotic protein critical for cell survival. XIAP binds caspase through the BIR domains to inhibit caspase apoptotic activity. XIAP also functions as an E3 ubiquitin ligase via the RING finger domain to degrade caspase through ubiquitination^11^,^12^. Our data show that the cleavage of procaspase-9 induced by OGD/reoxygenation is higher after co-transfected with wild-type procaspase-9 and wild-type XIAP than wild-type procaspase-9 and XIAP (C449G). Cys449 S-nitrosylation of XIAP is located in the RING finger domain; S-nitrosylation of Cys449 can downregulate the E3 ligase activity of XIAP and abrogate the XIAP-mediated inhibition of caspase activity and apoptosis^24^. So we presume that procaspase-9 transnitrosylates the RING finger domain of XIAP to inhibit its E3 ligase activity and promote apoptosis during cerebral ischemia-reperfusion or OGD/reoxygenation. However, Tsang and colleagues reported that the BIR domains of XIAP can be S-nitrosylated by GSNO *in vitro*, and S-nitrosylation of XIAP does not affect its E3 ligase activity but directly impairs its binding with caspase-3 to inhibit its anticaspase-3 activity^39^. However, the concentrations of exogenous NO required are much higher (500 μM) than those found in physiological states. We predict that very high concentrations of NO can S-nitrosylate non-selectively many other protein thiols, and endogenous NO more specifically can S-nitrosylate the RING finger domain of XIAP.

Considering the crucial role of the Trx system in regulating protein S-nitrosylation as mentioned above, we speculated that the Trx system may participate in the transnitrosylation from procaspase-9 to XIAP. As shown as Fig. 4, the denitrosylation of procaspase-9 and the S-nitrosylation of XIAP induced by cerebral ischemia-reperfusion were significantly suppressed through either inhibiting the enzyme activity or downregulating the expression of TrxR1. Hence, we confirm that the Trx1 system mediates the process by which procaspase-9 transnitrosylates XIAP during cerebral ischemia-reperfusion. However, the mechanism behind the Trx1 system mediating transnitrosylation from procaspase-9 to XIAP remains unknown. Mammalian Trx1 has five conserved cysteine residues: Cys32, 35, 62, 69, and 73. The free thiols of Cys32 and Cys35 selectively reduce disulfide bonds within target proteins, resulting in the formation of a disulfide bond between these cysteines and oxidized Trx1 (Trx1-S2). The Trx1 Cys32-Cys35 disulfide bond is reduced to free thiols by NADPH and TrxR to generate reduced Trx1 (Trx1- (SH)2), which in turn reduces disulfide bonds on proteins^22^. In addition, Cys62, 69, and 73 are associated with the modulation of protein transnitrosylation and denitrosylation^31^,^33^. Whether Trx1 catalyzes transnitrosylation or denitrosylation activities depends on its redox status. The denitrosylation of target proteins by Trx1 involves the reductive Cys32 and Cys35, and target NO is initially transferred onto the low pKa thiol of Cys32 before forming a disulfide bond with the vicinal thiol of Cys35, eventually resulting in the concomitant release of nitroxyl (HNO) or possibly transferring the NO intramolecularly to Cys62, 69, or 73 to form SNO-Trx1-S2^22^,^40^. Before catalyzing transnitrosylation activity, Trx1 itself needs to be nitrosylated first. Wu and colleagues found that only the oxidized form of Trx1 (Trx1-S2) could be nitrosylated on Cys73, which is responsible for Trx1-mediated transnitrosylation under physiological conditions. Trx1-S2 with SNO-Cys73 then could transnitrosylate targets such as procaspase-3^22^,^33^. Taken together, in this study we find a new mechanism for procaspase-9 transnitrosylating XIAP: after cerebral ischemia-reperfusion, the NO group of procaspase-9 is first transferred to the free thiol of Cys32 of Trx1- (SH)2 and subsequently transferred to Cys73 intramolecularly to form SNO-Trx1-S2. As a transnitrosylase, SNO-Trx1-S2 then transfers the NO group to XIAP.

XIAP can directly interact with caspases-3, -7, -9 and inhibit procaspase activity. Nakamura and his colleagues found that the SNO-caspase-3 transnitrosylates (transfers it’s NO group) to XIAP, forming SNO-XIAP, promotes accumulation of cleaved caspase-3 and thus promotes cell injury and death^10^,^11^,^24^. Here, our data confirmed that cerebral ischemia-reperfusion induced XIAP nitrosylation, procaspase-9 denitrosylation and cleavage. The potential interplay between caspase 3 and 9 via transnitrosylating to XIAP, leading to apoptosis, particularly in a cerebral ischemia-reperfusion event is underway in our lab. The results will be reported in the future.

## Additional Information

**How to cite this article**: Zhang, D. *et al*. Procaspase-9 induces its cleavage by transnitrosylating XIAP via the Thioredoxin system during cerebral ischemia-reperfusion in rats. *Sci. Rep*. **6**, 24203; doi: 10.1038/srep24203 (2016).
